# Assessment of the Effect of Methotrexate Therapy on Bone Metabolism in Patients with Rheumatoid Arthritis

**DOI:** 10.1007/s00005-015-0338-x

**Published:** 2015-04-03

**Authors:** Jerzy Świerkot, Katarzyna Gruszecka, Agnieszka Matuszewska, Piotr Wiland

**Affiliations:** 1 Department of Rheumatology and Internal Medicine, Wroclaw Medical University, Wrocław, Poland; 2 Department of Pharmacology, Wroclaw Medical University, Wrocław, Poland

**Keywords:** Dkk-1, Methotrexate, Osteoprotegerin, RANKL, Rheumatoid arthritis

## Abstract

Proinflammatory cytokines and growth factors, which regulate mutual interactions between immune system cells and bone tissue cells, play a major role in the formation of bone changes in rheumatoid arthritis (RA). The aim of the work was to assess serum concentration of osteoprotegerin (OPG), RANKL, Dkk-1 and sclerostin in RA patients compared to a control group and to analyze changes of these concentrations during methotrexate (MTX) therapy. Patients enrolled in the study were 30 women of Caucasian origin aged 30–74 years with RA. Patients with active form of the disease were administered recommended doses of MTX for at least 6 months. The study group was divided into subgroup I—patients with improvement; and subgroup II—patients with no improvement. The control group consisted of 12 healthy women in the age of 41–73. Before MTX therapy, RA patients had higher levels of RANKL (644.97 ± 477.13 vs. 255.19 ± 130.26 pmol/l), lower values of OPG/RANKL (0.01 ± 0.0101 vs. 0.02 ± 0.0078) and higher levels of Dkk-1 protein (1821.32 ± 1060.28 vs. 548.52 ± 36.35 pg/ml) compared to the control group. In the analyzed group of patients (all patients receiving MTX regardless of responder non responder status) after 6 months of therapy, a statistically significant increase in the ratio of OPG/RANKL was found (0.0118 ± 0.0102 vs. 0.0141 ± 0.0118; *p* = 0.02). The index value of OPG/RANKL differed significantly depending on the resultant effect of treatment (0.01702 ± 0.01274 in the subgroup of improvement vs. 0.00675 ± 0.00289 in the subgroup without improvement). The difference in the mean concentrations of Dkk-1 before and after treatment with MTX between subgroups I and II was statistically significant (*p* = 0.002). In subgroup I, mean concentration of Dkk-1 decreased after 6 months of treatment with MTX (2054.72 ± 1004.74 vs. 1831.70 ± 851.70 pg/ml); while in subgroup II, the mean concentration of Dkk-1 increased (1214.48 ± 738.32 vs. 2275.01 ± 1385.23 pg/ml). There were no statistically significant changes in the mean concentrations of sclerostin before and after treatment with MTX (in whole group treatment with MTX, in subgroup I, and in subgroup II). The results confirm the presence of disorders of bone metabolism in patients with RA. Treatment with MTX affects the value of the ratio of OPG/RANKL and concentration of Dkk-1.

## Introduction

Rheumatoid arthritis (RA) is a chronic disease of autoimmune origin that, when improperly treated, causes disability in most cases. It affects 1 % of adults, predominantly women. It is associated with synovial inflammation with accompanying cartilage damage and bone lesions (Scott et al. 2010). The radiological features include periarticular osteopenia, erosions, joint space narrowing and deformity such as subluxation and dislocation (Brown 2013).

However, the mechanism of these lesions is not fully understood. It has been demonstrated that there is a significant role of proinflammatory cytokines and growth factors that regulate mutual interactions between immune system cells and bone tissue cells: osteoclasts, osteoblasts and osteocytes. Osteoclasts are responsible for bone resorption and osteoblasts are responsible for the synthesis and mineralization of bone (Neve et al. 2011).

Bone destruction is controlled by the complex interplay between three molecules essential for bone biology: receptor activator of nuclear factor κB ligand (RANKL), RANK and osteoprotegerin (OPG) (Revu et al. 2013; Schett et al. 2005). The connection of RANKL with its receptor RANK induces the process of formation of osteoclasts (proliferation, differentiation, functioning) and inhibits osteoclast apoptosis (Kmieć and Sokołowska 2007; Vis et al. 2013). Mice with a disrupted RANKL gene show severe osteopetrosis and completely lack osteoclasts (Kong et al. 1999). Catabolic effects of RANKL are inhibited by OPG, thereby preventing the activation of its receptor RANK (Danks and Takayanagil 2013; Sinningen et al. 2012). During physiological bone remodeling, the ratio of RANKL to OPG is balanced (Sinningen et al. 2012).

The Wnt/β-catenin pathway participates in the regulation of osteoblasts. It affects the proliferation, maturation, function and apoptosis of these cells (Kryśkiewicz and Lorenc 2010). The presence of strong inhibitors of Wnt/β-catenin protein [Dickkopf-1 (Dkk-1), sclerostin] results in the inhibition of bone formation (Vis et al. 2013).

Methotrexate (MTX) is a first-line drug in RA (Smolen et al. 2010). It is one of the disease-modifying anti-rheumatoid drugs (DMARDs). It slows down articular damage in RA patients (Kucharz 2011). MTX inhibits dihydrofolate reductase activity, thus blocking synthesis of purine nucleotides and thymidylates, which are essential for nucleic acid synthesis and repair, and cell replication (Orzechowska-Juzwenko 2001). The question whether the main mode of action of the drug is immunosuppressive, immunomodulating, cytostatic or anti-inflammatory is still being studied. One of the significant modes of MTX action is its effect on inflammation inhibition through increase of extracellular adenosine concentration.

Studies on animal models demonstrate that high doses of MTX may damage bone progenitor cells and inhibit proliferation of osteoblasts and intensify bone resorption (Fan et al. 2011). Use of MTX in patients with malignancies was associated with bone mass decrease and osteoporosis (Stava et al. 2009). However, there still remain doubts about the adverse effect of MTX on bone tissue metabolism in RA. Much lower doses are used in these cases. Only isolated papers on bone metabolism regulation changes in RA patients are available in the literature.

In the active RA occurs the severity of bone resorption and decrease bone formation. El Miedany et al. (1998) observed after treatment with MTX reduction in bone resorption which may be due to the reduction of disease activity. Revu et al. (2013) observed that MTX decreased synovial cellularity in RA. Changes in cellularity were accompanied by decrease in local expression of RANKL and the RANKL/OPG ratio. They did not observe change in the expression of OPG (Revu et al. 2013).

The aim of the work was to assess serum concentration of OPG, RANKL, Dkk-1 and sclerostin in RA patients compared to a control group and to analyze changes of these concentrations during MTX therapy and in relation to achieved response to therapy.

## Materials and Methods

### Characteristics of Patients

Patients enrolled in the study were 30 women of Caucasian origin aged 30–74 years with an established diagnosis of RA according to American College of Rheumatology (ACR) criteria. In connection with literature data showing the differences in etiology of osteoporosis in men and women (the influence of sex hormones, other growth rate of bone loss, reaching another peak bone mass), only women were enrolled in the study. Patients with active form of the disease were administered recommended doses of MTX for at least 6 months. The study group was divided into subgroup I—patients with improvement, and subgroup II—patients with no improvement.

Three patients were excluded from the analysis due to irregular reporting to follow-up visits and incomplete data. Twenty-seven women aged 30–74 years (mean 54.7 years, SD *s* = 12.36, median *x*_med_ = 55) were qualified for the final analysis. 73 % of women in the study group were postmenopausal. Mean disease duration was 4.75 years—in 59 % of the patients it was for 2 years. In 12 patients (44 %), disease duration was 12 months; in 4 patients (15 %), it was between 12 and 24 months; while in 11 patients (41 %), it was over 24 months. To determine the severity of bone lesions according to the Steinbrocker classification, patients underwent X-ray of hands (Steinbrocker et al. 1949). In terms of severity of radiological changes, the patients were divided into two groups: the first comprising patients with stages I and II changes, the second comprising patients with stages III and IV changes. According to this classification, the first-stage RA is characterized by synovitis, or an inflammation of the synovial membrane, causing swelling of involved joints and pain upon motion. However, there is no X-ray evidence of joint destruction, with the exception of swelling of soft tissues or early stages of osteoporosis. In stage II, there is a spread of inflammation in synovial tissue, affecting joint cavity space across joint cartilage. This inflammation will gradually result in a destruction of cartilage, accompanied by a narrowing of the joint. Severe RA, stage III, is marked by the formation of pannus in the synovium. Loss of joint cartilage exposes bone beneath the cartilage. These changes will become evident on X-ray, along with erosions and signs of deformation. Stage IV is called terminal or end-stage RA. The inflammatory process has subsided and formation of fibrous tissue and/or fusing of bone results in ceased joint function. Rheumatoid nodules may also be present in patients in stage IV of the disease.

The following inclusion criteria were accepted: consent to participate in the study; confirmed RA based on 1987 classification criteria of the ACR; active form of the disease—disease activity score based on erythrocyte sedimentation rate and an evaluation of 28 joints (DAS28) ≥ 4.0 (26 of patients had DAS28 > 5.1); no use of MTX during the last 1 year; age over 18 years.

There were the following exclusion criteria: pregnancy or breastfeeding; coexistence of other systemic diseases of connective tissue besides RA; clinically significant impairment of hepatic and renal function; alcohol abuse; infection with hepatotropic viruses; infections resistant to therapy; ongoing history of cancer if no cure was achieved; uncontrolled diabetes; patient unwilling or unable to cooperate. Patients were administered with recommended doses of MTX for at least 6 months. Patients were starting therapy with 15 mg MTX once a week, and the dose could be increased to 25 mg once weekly (mean dose 15.4 mg/week). The patients were allowed to continue treatment with other DMARDs, glucocorticoids, and/or non-steroidal anti-inflammatory drugs, if the treatment regimens were not modified 4 weeks before the study. Fifty-two percent of patients used prednisone at a mean dose of 7.8 mg/day (4–10 mg/day). Prednisone doses were constant for at least 8 weeks before the start of the study and during the study.

To examine the response to MTX therapy, blood samples, laboratory data, and clinical data were collected at baseline (prior to MTX therapy), 2, 4 and 6 months post treatment. Clinical evaluation was based on medical history, number of painful and swollen joints, pain intensity assessed by the patient on a 100-mm visual analog scale and laboratory tests (ESR, CRP). The parameters allowed determination of improvement according to the criteria based on DAS28 suggested by the European League Against Rheumatism (EULAR). According to the EULAR definition, patients are classified as good, moderate, or non-responders, using the individual amount of change in the DAS28 (ΔDAS28) and DAS28 values at 6 months. A good responder is classified if ΔDAS28 ≥ 1.2 and DAS28 at 3 or 6 months ≤3.2; moderate responders are patients with (ΔDAS28 ≥ 1.2 and DAS28 at 3 or 6 months ≤5.1 and >3.2) or (0.6 < ΔDAS28 ≤ 1.2 and DAS28 at 3 or 6 months ≤5.1). Patients are classified as non-responders if they do not fall into any of these categories. Moreover, in some analyses, a comparison was made between patients who had a good or moderate response (subgroup I) and patients with no response (subgroup II) which was due to the limited number of patients in each group.

Safety of MTX therapy was analyzed on the basis of medical history, physical examination, and selected laboratory tests [including blood cell count, aspartate aminotransferase (AST), alanine aminotransferase (ALT), creatinine, urea levels, and urinalysis]. The clinical and laboratory tests were completed before the start of the therapy and in months 2, 4 and 6 of the follow-up. The concentrations of modulators of bone metabolism (OPG, RANKL, Dkk-1 and sclerostin) were determined at baseline and after 6 months. Designations were performed by ELISA using kits: OPG (DRG Instruments GmbH, Germany), sRANKL (DRG Instruments GmbH, Germany), sclerostin (TECOmedical Group, Switzerland), human Dkk-1 (Assay Design Inc., USA). The patients’ characteristics are presented in Table 1. All the patients provided written informed consent. The study was approved by the Wroclaw Medical University Ethics Committee.

**Table 1:** Characteristics of rheumatoid arthritis (RA) patients at baseline and after 6 months of MTX therapy

	Baseline	After 6 months of MTX therapy
RA patients (*n*)	27	27
Females (%)	100	100
Age, mean (range)	54.7 (30–74)	
Disease duration (years), mean (range)	4.75 (0.25–12)	
Disease activity score DAS28, mean (range)	6.0 (4.9–7.9)	4.5 (3.2–6.4)
Tender joint count, mean (range)	12 (4–26)	6 (1–16)
Swollen joint count, mean (range)	7 (3–13)	3 (0–10)
Erythrocyte sedimentation rate (mm/h), mean (range)	48 (16–94)	35 (12–78)
Patient pain (mm), mean (range)	63 (27–85)	41 (12–70)

The control group consisted of 12 healthy women aged 41–73 (mean age 56.9, SD 11.06, median 56). Seventy-five percent of women in the control group were postmenopausal.

The compared groups of women (study and control) were consistent in relation to age, which was verified with the parametric Student’s *t* test following normality check with the Shapiro–Wilk test.

### Statistical Analysis

The results are presented as mean ± SD. Normality of distribution was verified with Shapiro–Wilk normality test. If normal distribution was proofed, the comparison of the groups was performed with parametric Student’s *t* test. If the distribution of markers was significantly different from normal distribution, a non-parametric Mann–Whitney *U* test was used for comparison. When comparing the results before and after treatment tests for dependent observations were used. In case of normally distributed variables Student’s *t* test was applied. In other cases differences were check with a non-parametric sign test. The results were treated as significant if *p* value was less than 0.05. The statistical analysis was performed with Statistica.

## Results

### Comparative Analysis of Bone Metabolism Regulator Concentrations in RA and Control Group

**Fig. 1:**
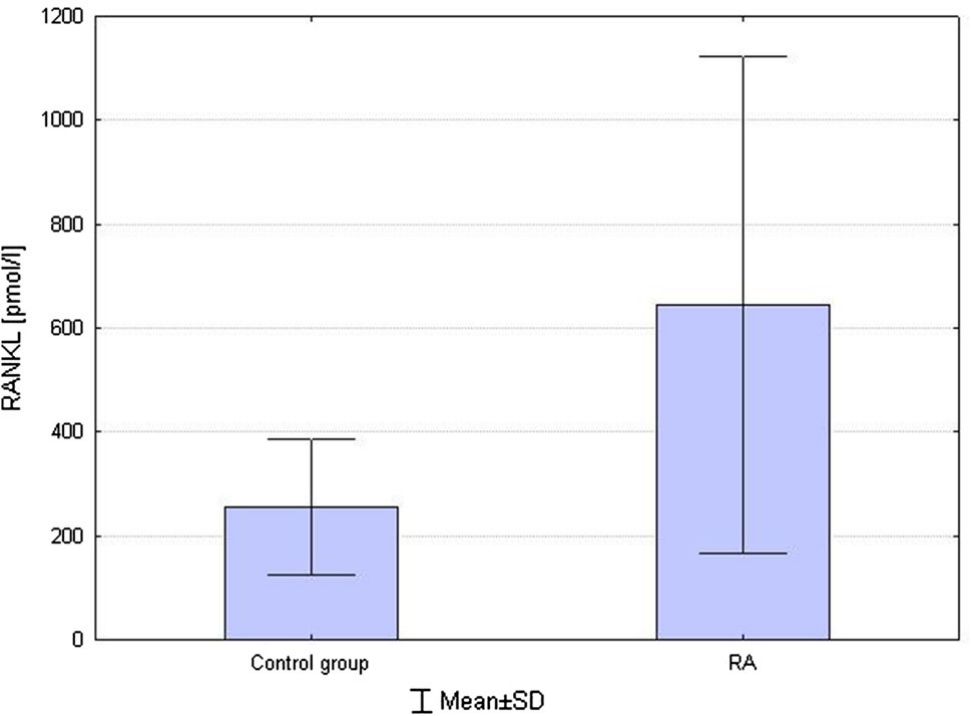
Comparison of RANKL levels between RA and control group. Higher RANKL concentrations compared to control group (*p* = 0.003)
- lower values of OPG/RANKL ratio compared to control group (0.01124 ± 0.0101 vs. 0.01885 ± 0.0078, respectively; *p* = 0.007) (Fig. 2),

In the current study, the following differences were statistically significant in RA patients before starting on MTX:

- higher RANKL concentrations compared to control group (644.97 ± 477.13 vs. 255.19 ± 130.26 pmol/l, respectively; *p* = 0.003) (Fig. 1),

**Fig. 2:**
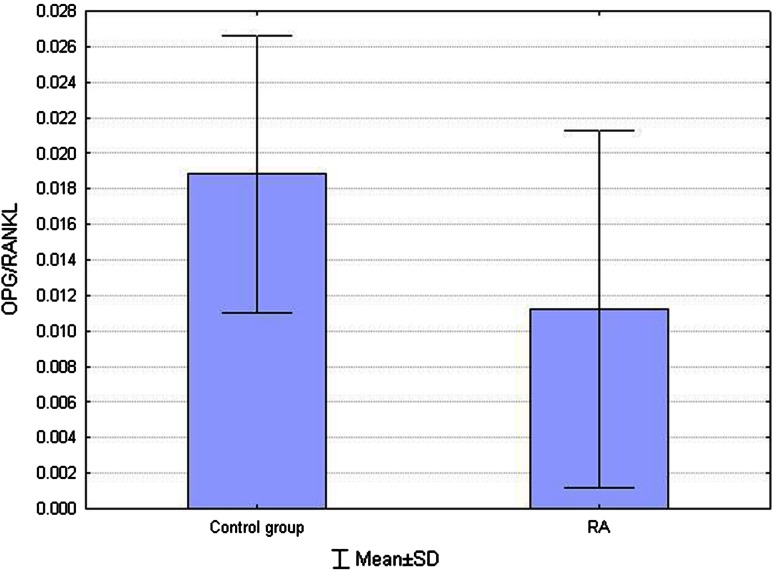
Comparison OPG/RANKL ratio between RA and control group. Lower values of OPG/RANKL ratio compared to control group (*p* = 0.007)
- higher concentration of Dkk-1 compared to control group (1821.32 ± 1060.28 vs. 548.52 ± 36.35 pg/ml, respectively; *p* = 0.0003) (Fig. 3).

**Fig. 3:**
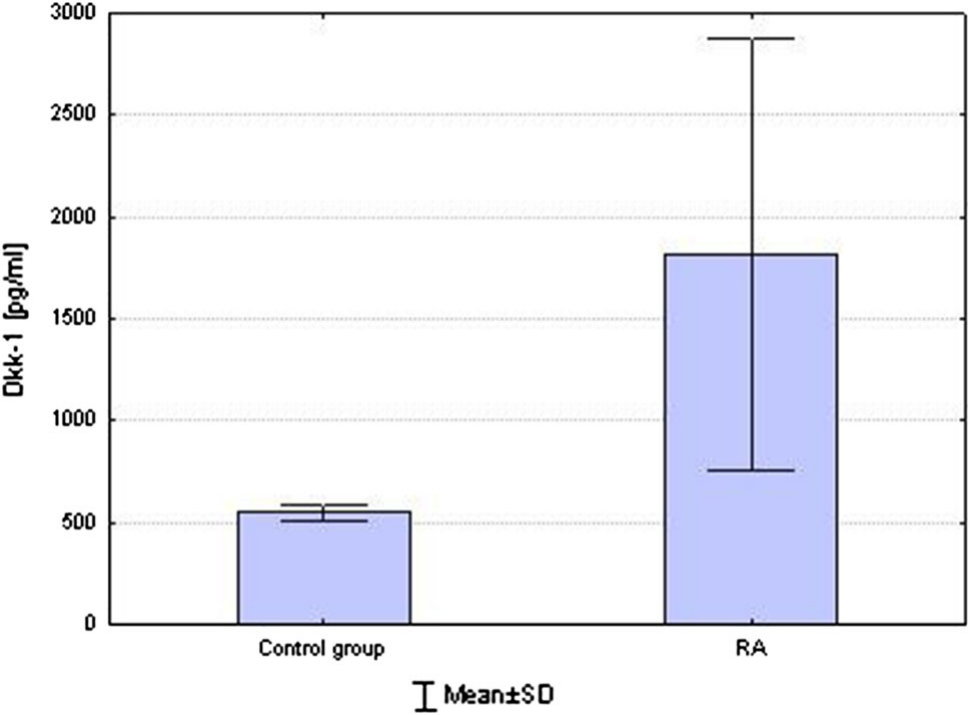
Comparison concentration of Dkk-1 between RA and control group. Higher concentration of Dkk-1 compared to control group (*p* < 0.001)

Osteoprotegerin levels and sclerostin levels, however, were similar in both groups (OPG 4.17 ± 1.62 vs. 4.13 ± 0.93 pmol/l, sclerostin 0.40 ± 0.26 vs. 0.35 ± 0.17 ng/ml, respectively, *p* > 0.05).

### Assessment of Bone Metabolism Regulator Concentrations in RA Patients During MTX Therapy

**Fig. 4:**
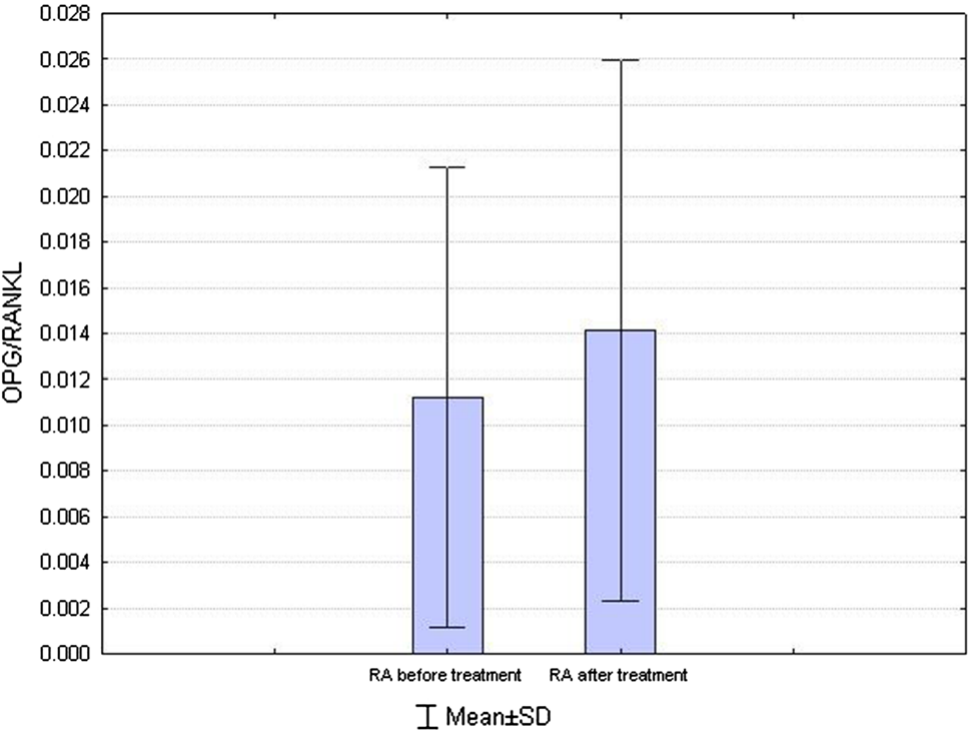
Comparison of the ratio of OPG/RANKL in patients with RA before (OPG/RANKL 1) and after (OPG/RANKL 2) treatment with MTX. After 6 months of therapy, a statistically significant increase in the ratio of OPG/RANKL was observed (*p* = 0.02)

In the analyzed group of patients after 6 months of therapy, a statistically significant increase in the ratio of OPG/RANKL was observed (0.0118 ± 0.0102 vs. 0.0141 ± 0.0118; *p* = 0.02) (Fig. 4). Concentrations of RANKL (644.97 ± 477.13 vs. 526.32 ± 378.95 pmol/l), sclerostin (0.40 ± 0.26 vs. 0.38 ± 0.26 ng/ml) and Dkk-1 (1821.32 ± 1060.28 vs. 1954.85 ± 1002.32 pg/l) did not change significantly.

### Assessment of Bone Metabolism Regulator Concentrations in RA Patients in Relation to Response to MTX Therapy

The study group of RA patients was subdivided in relation to response to treatment (subgroup I—good or moderate response, subgroup II—no response). Subgroup I consisted of 20 patients (14 patients with good improvement, 6 patients with moderate improvement) and subgroup II—7 patients with no improvement.

**Fig. 5:**
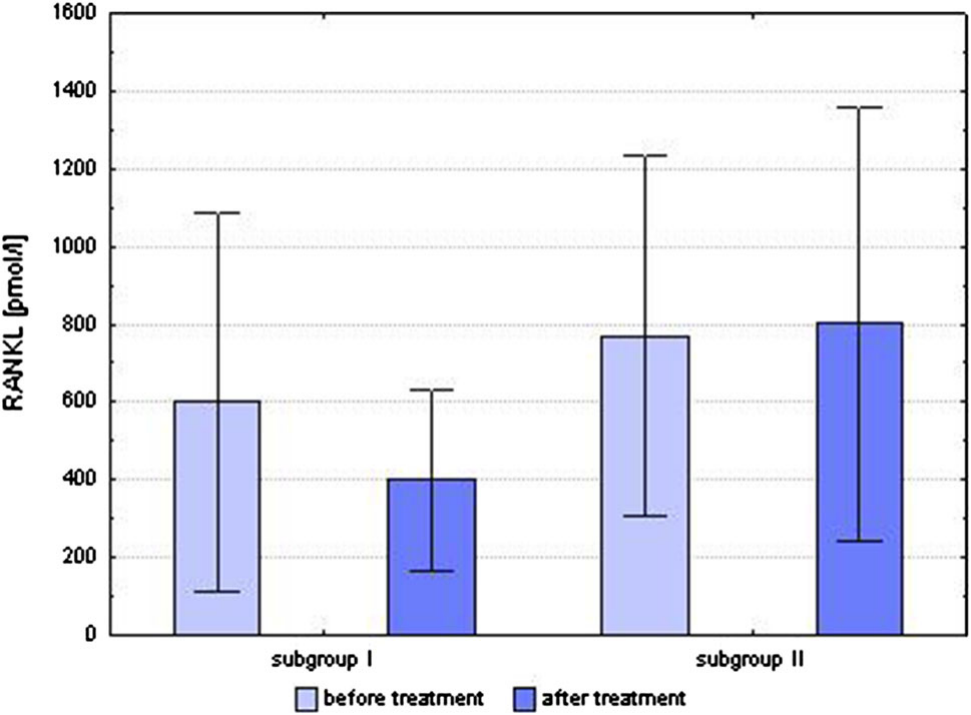
Comparison of the level of RANKL in patients with improvement (subgroup I) and patients with no improvement (subgroup II) after 6 months of treatment with methotrexate. There was found a statistically significant difference between mean values of the concentrations of RANKL between subgroups I and II after treatment with MTX (*p* < 0.05)

In the present study, the mean concentration of RANKL in the subgroup of improvement (subgroup I) decreased after 6 months of treatment with MTX (601.01 ± 486.48 vs. 401.15 ± 233.42 pmol/l), and in the subgroup with no improvement (subgroup II) increased (770.55 ± 460.66 vs. 801.45 ± 557.79 pmol/l). There was found a statistically significant difference between mean values of the concentrations of RANKL between subgroups I and II after treatment with MTX (401.15 ± 233.42 vs. 801.45 ± 557.79 pmol/l; *p* = 0.004) (Fig. 5). In subgroup I, there was a greater increase in mean concentration of OPG than in subgroup II (0.27 ± 0.73 vs. 0.04 ± 1.15 pmol/l), but this difference was not statistically significant.

**Fig. 6:**
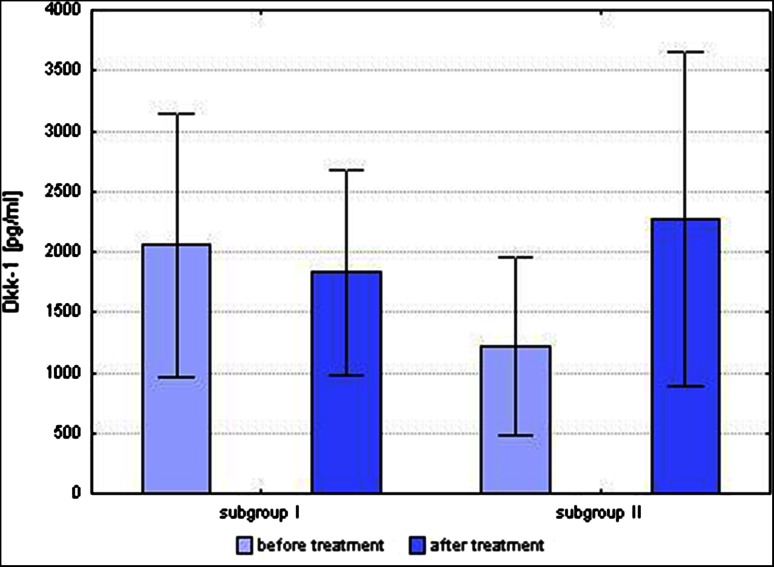
Comparison of the level of Dkk-1 in patients with improvement (subgroup I) and patients with no improvement (subgroup II) after 6 months of treatment with MTX. In subgroup I mean concentration of Dkk-1 decreased after 6 months of treatment with MTX, while in subgroup II the mean concentration of Dkk-1 increased (*p* < 0.01)

Before starting treatment with MTX, the ratio of OPG/RANKL was comparable between subgroups I and II (0.01267 ± 0.01118 vs. 0.00716 ± 0.00402). However, the value of the ratio of OPG/RANKL after treatment with MTX between subgroups I and II differed significantly (0.01702 ± 0.01274 vs. 0.00675 ± 0.00289; *p* = 0.03). The difference in the mean concentrations of Dkk-1 before and after treatment with MTX between subgroups I and II was statistically significant (*p* = 0.002) (Fig. 6). In subgroup I, mean concentration of Dkk-1 decreased after 6 months of treatment with MTX (2054.72 ± 1004.74 vs. 1831.70 ± 851.70 pg/ml); while in subgroup II, the mean concentration of Dkk-1 increased (1214.48 ± 738.32 vs. 2275.01 ± 1385.23 pg/ml). There were no statistically significant changes in the mean sclerostin concentrations before and after treatment in subgroup I (0.43 ± 0.29 vs. 0.32 ± 0.12 ng/ml) or subgroup II (0.41 ± 0.28 vs. 0.32 ± 0.09 ng/ml).

### Assessment of Bone Metabolism Regulator Concentrations in RA Patients Depending on the Severity of Bone Lesions According to the Steinbrocker Classification

The study group of RA patients was subdivided in relation to the Steinbrocker classification. In the group of patients with grades I and II changes (combined group) in the Steinbrocker classification compared to patients with grades III and IV changes (combined group), we found no statistically significant difference in the concentrations of RANKL, OPG, Dkk-1, sclerostin, or ratio of OPG/RANKL.

This concerned both before (respectively, RANKL 572.36 ± 433.45 vs. 767.31 ± 544.48 pmol/l, OPG 4.34 ± 1.56 vs. 3.88 ± 1.77 pmol/l, Dkk-1 2016.57 ± 1172.80 vs. 1514.51 ± 844.44 pg/ml, sclerostin 0.41 ± 0.28 vs. 0.39 ± 0.22 ng/ml, OPG/RANKL 0.01 ± 0.01 vs. 0.01 ± 0.01) and after treatment (respectively, RANKL 531.36 ± 464.41 vs. 481.00 ± 209.43 pmol/l, OPG 4.68 ± 1.69 vs. 4.26 ± 1.80 pmol/l, Dkk-1 2153.53 ± 1161.09 vs. 1642.63 ± 643.03 pg/ml, sclerostin 0.43 ± 0.26 vs. 0.36 ± 0.22 ng/ml, OPG/RANKL 0.02 ± 0.01 vs. 0.01 ± 0.01). In any case, the *p* value does not exceed the predetermined level of significance 0.05.

## Discussion

Osteoclasts originate from the monocyte–macrophage lineage under modulation of cytokines: macrophage colony-stimulating factor (M-CSF) and RANKL (Maeda et al. 2013). M-CSF modulates proliferation, differentiation and fusion of precursors and at later differentiation stages bone resorbing activity (Braun and Zwerina 2011). RANKL is a key mediator in differentiation, fusion and activation of osteoclasts (Kryśkiewicz and Lorenc 2010; Nemeth et al. 2011). The connection of RANKL with the RANK stimulates osteoclastogenesis. On the other hand, binding of RANKL with OPG, which is a soluble competitive receptor, inhibits osteoclast maturation (Kmieć and Sokołowska 2007; Kryśkiewicz and Lorenc 2006).

In RA patients, bone metabolism disorders occur. Bone resorption is increased. Enhanced proliferation and activity of osteoclasts cause bone erosions and bone mass loss. Physiologically, RANKL is synthesized by osteoblasts and stromal cells. In inflammatory conditions, under presence of cytokines (TNF-α, IL-1), it can also be synthesized by T cells, fibroblasts, synoviocytes, monocytes/macrophages and activated B cells (Gravallese et al. 2000; Kmieć and Sokołowska 2007). In the current study, higher serum RANKL and a lower OPG/RANKL ratio were found in RA patients compared to control subjects. This is consistent with results obtained by other authors (Ellabban et al. 2012; Skoumal et al. 2005; Xu et al. 2012).

Osteoblasts participate in bone formation. Proliferation, function and apoptosis of osteoblasts are regulated by the Wnt/β-catenin pathway. Wnt family protein is a ligand for a Frizzled receptor and triggers it resulting in stabilization of the signal transduction proteins responsible for key gene expression, that is, β-catenin (Pawlak-Buś and Leszczyński 2011). Natural inhibitors of the Wnt/β-catenin pathway are Dkk-1 and sclerostin (Goldring et al. 2013).

Dkk-1 protein inhibits bone formation (Goldring et al. 2013). It also has an impact on the severity of the process of bone resorption (Diarra et al. 2007). TNF-α induced Dkk-1 expression on synovial fibroblasts (Diarra et al. 2007; Goldring et al. 2013). Local production of Dkk-1 in synovial membrane is related to its serum level increase (Diarra et al. 2007). In the current study, statistically significantly higher levels of Dkk-1 were observed in patients with RA compared to the control group. This is consistent with the results obtained by Wang et al. (2011).

Still the MTX effect on bone metabolism is not entirely clear. Literature data report a decrease or no change of bone tissue resorption markers during MTX therapy. Torikai et al. (2006) found a decrease of N-terminal crosslinked telopeptide of chain α of type I collagen, and El Miedany et al. (1998) observed a decrease in deoxypyridinoline (DPD) level during therapy. However, Mianur et al. (2002) observed no statistically significant changes of DPD levels in RA patients treated with MTX. In the present study, there was an increase in the ratio of OPG/RANKL after 6 months of treatment with MTX. The index value of OPG/RANKL is an important factor in regulating the severity of the process of bone resorption (Haynes et al. 2001). Predominance of RANKL over OPG increases bone resorption, while predominance of OPG over RANKL reduces such activity (Kryśkiewicz and Lorenc 2006).

In animal RA models, Dkk-1 inhibition by a specific antibody targeted against it was associated with osteogenesis improvement (Diarra et al. 2007; Goldring et al. 2013). El Miedany et al. (1998) found increased levels of an osteogenesis marker (bone alkaline phosphatase) in RA patients after 3 months of MTX therapy. In the present study, no statistically significant change of Dkk-1 protein level after MTX therapy was observed in the whole RA group. However, in patients with a good response to therapy the Dkk-1 level decreased, and in patients with no response it increased. Dkk-1 concentration in serum correlates with disease activity (Diarra et al. 2007). Therefore, effective therapy with MTX (15–25 mg/week) in RA patients appears to positively influence bone tissue metabolism through disease activity control. Patients not responding to therapy deserve particular attention, as they require therapy intensification.

We are aware of the limitations of our study, which arise from the small study group, observation of only women mainly in the postmenopausal period and 50 % of patients used prednisone in constant dose for at least 8 weeks before the start of the study and during the study. To confirm the results, we plan to continue the studies on a larger group of patients including also other groups of patients with RA.

In conclusion, the results confirm the presence of disorders of bone metabolism in patients with RA. The administration of MTX affects the value of the ratio of OPG/RANKL and concentration of Dkk-1. It is important for inhibiting the progression of bone loss to have an effective therapy in RA patients, decreasing the inflammatory activity. Particular attention should be paid to patients not responding to treatment, who should receive intensified therapy.
